# Incidence, risk factors and outcomes of acute kidney injury among COVID-19 patients: A systematic review of systematic reviews

**DOI:** 10.3389/fmed.2022.973030

**Published:** 2022-11-04

**Authors:** Tauqeer Hussain Mallhi, Yusra Habib Khan, Abdulaziz Ibrahim Alzarea, Faiz Ullah Khan, Nasser Hadal Alotaibi, Abdullah Salah Alanazi, Muhammad Hammad Butt, Ahmed D. Alatawi, Muhammad Salman, Sami I. Alzarea, Ziyad Saeed Almalki, Mansoor A. Alghazi, Majed Ahmed Algarni

**Affiliations:** ^1^Department of Clinical Pharmacy, College of Pharmacy, Jouf University, Sakaka, Saudi Arabia; ^2^Health Sciences Research Unit, Jouf University, Sakaka, Saudi Arabia; ^3^Department of Pharmacy Administration and Clinical Pharmacy, School of Pharmacy, Xi’an Jiaotong University, Xi’an, China; ^4^Department of Medicinal Chemistry, Faculty of Pharmacy, Uppsala University, Uppsala, Sweden; ^5^Institute of Pharmacy, Faculty of Pharmaceutical and Allied Health Sciences, Lahore College for Women University, Lahore, Pakistan; ^6^Department of Pharmacology, College of Pharmacy, Jouf University, Sakaka, Saudi Arabia; ^7^Department of Clinical Pharmacy, College of Pharmacy, Prince Sattam Bin Abdulaziz University, Al-Kharj, Saudi Arabia; ^8^Department of Pharmaceutical Care, King Abdulaziz Medical City, Riyadh, Saudi Arabia; ^9^Department of Clinical Pharmacy, College of Pharmacy, Taif University, Taif, Saudi Arabia

**Keywords:** COVID-19, SARS-CoV-2, coronavirus, acute kidney injury, complications, mortality, risk factors

## Abstract

The COVID-19 associated acute kidney injury (CAKI) has emerged as a potential intricacy during the management of patients. Navigating the rapidly growing body of scientific literature on CAKI is challenging, and ongoing critical appraisal of this complication is essential. This study aimed to summarize and critically appraise the systematic reviews (SRs) on CAKI to inform the healthcare providers about its prevalence, risk factors and outcomes. All the SRs were searched in major databases (PubMed, EMBASE, Web of Science) from inception date to December 2021. This study followed SR of SRs methodology, all the records were screened, extracted and subjected to quality assessment by assessing the methodological quality of systematic reviews (AMSTAR-2). The extracted data were qualitatively synthesized and tabulated. This review protocol was registered in PROSPERO (CRD42022299444). Of 3,833 records identified; 42 SRs were included in this overview. The quality appraisal of the studies showed that 17 SRs were of low quality, while 8 moderate and 17 were of high-quality SRs. The incidence of CAKI ranged from 4.3% to 36.4% in overall COVID-19 patients, 36%–50% in kidney transplant recipients (KTRs), and up to 53% in severe or critical illness. Old age, male gender, cardiovascular disease, chronic kidney disease, diabetes mellitus and hypertension were frequently reported risk factors of CAKI. The need of renal replacement therapy (RRT) was up to 26.4% in overall COVID-19 patients, and 39% among those having CAKI. The occurrence of acute kidney injury (AKI) was found independent predictor of death, where mortality rate among CAKI patients ranged from 50% to 93%. This overview of SRs underscores that CAKI occurs frequently among COVID-19 patients and associated with high mortality, need of RRT and adverse outcomes. However, the confidence of these results is moderate to low which warrants the need of more SRs having established methodological standards.

**Systematic review registration:**

[https://www.crd.york.ac.uk/prospero/display_record.php?RecordID=299444], identifier [CRD42022299444].

## Introduction

The clinical spectrum of COVID-19 is typically manifested by respiratory intricacies including dyspnea, alveolar injury and respiratory failure. However, the rapid geographical distribution of infection showed the involvement of various other organs during the disease course. Acute kidney injury (AKI) has emerged as one of the most common atypical complications of COVID-19 infection. Its pathogenesis is attributed to the cytokine storm, fluid loss associated with hypovolemia (due to high-grade pyrexia and tachypnea), acute respiratory distress syndrome (ARDS), and direct viral invasion specifically into intrinsic renal cells (1, 2). SARS-CoV-2 is a cytopathic virus that enters the renal cells through ACE2. Since spike protein on the viral surface is activated by a cellular transmembrane serine protease (TMPRSSs), ACE2 is expressed along with TMPRSSs in proximal straight tubule cells and podocytes, thereby exposing the kidney to SARS-CoV-2 (3).

The growing body of evidence suggests that COVID-19 associated acute kidney injury (CAKI) occurs in a considerable number of patients and is accompanied by the increased duration of hospital stay, healthcare cost, renal replacement therapy (RRT), and mortality (1, 2, 4). Researchers around the globe responded to this issue by publishing an impressive number of reports on the incidence, risk factors and clinical outcomes of CAKI. Despite an increasing number of studies relating to various aspects of CAKI and its prognosis; discrepancies in size, methodologies, and research focus as well as regional differences in these studies call for a comprehensive systematic review and analysis of reports to date. Moreover, the quality and reproducibility of these evidences have become an area of concern for healthcare professionals across the globe. Systematic reviews are considered a cornerstone of evidence-based medicine and reflect the current scientific knowledge. Systematic reviews hold a highest level in the hierarchy of evidence, aiding healthcare practitioners to have informed decisions on a specific topic (5). The large volume of studies on renal involvements in COVID-19 has encouraged the researchers to systematically synthesize the findings of these studies, resulting in a large number of systematic reviews on this topic. Navigating the rapidly growing body of scientific literature on CAKI is challenging, and ongoing critical appraisal of this complication is essential.

Systematic Review of Systematic Reviews (SR of SRs) is a subtype of Overview of Systematic Reviews, which allows performing a comprehensive review of the highest level of evidence from already synthesized SR-level data with the end product ready to be used by the clinicians and policy-makers (6, 7). To the best of our search, the incidence of CAKI, risk factors and clinical outcomes have been discussed in 42 SRs. Considering the high number of SRs, it is imperative to perform an overview so existing literature could be identified and organized to underscore the areas of priority in decision making. Moreover, this overview will provide a composite draft of recent advancements in AKI among COVID-19 patients. In this context, this study aimed to summarize and critically appraise the SRs on CAKI to ascertain its incidence, risk factors, need for RRT, associated mortality, and other adverse outcomes.

## Materials and methods

### Ethics

This study is exempted from ethical approval because it involves qualitative synthesis of publicly available data.

### Review question

This SR of SRs was intended (1) to summarize and critically appraise the SRs on AKI during the COVID-19 infection, and (2) to ascertain the reported incidence of CAKI, contributing risk factors, need for RRT, and mortality among COVID-19 patients.

### Review protocol registration

The protocol of the current review is registered in PROSPERO (Registration no. CRD42022299444).

### Study design

This study followed the SR of SRs methodology, which is a subtype of an overview of SRs. This review adheres to the Preferred Reporting Items for Systematic Reviews and Meta-analyses (PRISMA) statement (8), and Preferred Reporting Items for Overview of Systematic Reviews (PRIO-harms) (7). The results of existing SRs on incidence, risk factors, and clinical outcomes of CAKI are synthesized, without re-synthesizing the primary studies.

### Main outcomes

The primary outcomes evaluated were; (1) incidence of AKI in COVID-19, (2) risk factors or predictors of CAKI, (3) need for RRT among COVID-19 patients, with or without AKI (4) impact of CAKI on mortality, and (5) impact of CAKI on other adverse clinical outcomes i.e., admissions to intensive care units (ICU), the incidence of severe infections, and graft loss among transplant recipients. The incidence of CAKI was presented in proportion (%), risk factors or predictors of CAKI are indicated as odds ratio (OR), Q, or mean differences (SMD, WMD), need for RRT among COVID-19 patients in OR and proportion (%), and mortality in OR, risk ratio (RR), and proportion (%).

### Information sources and search strategy

A systematic search was performed in databases (PubMed, Scopus, ProQuest, PMC), review registries (PROSPERO and CENTRAL) and Google scholar from the inception date to December 2021. The search strategy used in the current study is described in Table 1 and in Supplementary File. The PubMed function “related articles” was used to extend the search. PI (E) CO formula was considered for framing the research question and inclusion/exclusion criteria.

**TABLE 1:** Search strategy and PECOS framework.

Search strategy	
**Databases**	PubMed, Scopus, ProQuest, PMC, CENTRAL, PROSPERO, and Google Scholar
Data duration	1 November 2019 to 30 December 2021
#1	“2019-nCoV” or “SARS-CoV-2” or “COVID-19” or “Coronavirus Disease”
#2	“Acute kidney injury” or “acute renal failure” or “acute renal injury” or “acute kidney failure” or “Kidney injury” or “renal impairment” or “kidney impairment”
#3	“Review,” “systematic review” or “meta-analysis” or “meta regression
Search	1 AND 2 AND 3

**PECOS framework**	

Population	Patients with confirmed diagnosis of COVID-19
Exposure	COVID-19 Patients with a confirmed diagnosis of Acute Kidney Injury (AKI)
Comparison	COVID-19 patients without Acute Kidney Injury (AKI)
Outcomes	Prevalence of AKI, factors contributing to AKI among COVID-19 patients and impact of AKI on prognosis (need for RRT, mortality, recovery)
Study design	Systematic or meta-analysis, or both regardless of type of manuscript

### Study selection

Two reviewers independently searched databases by adopting the search strategy (Table 1). The relevancy of each study was evaluated initially through screening of title and abstract. Only studies providing information on incidence, risk factors, and prognosis or outcomes of AKI among COVID-19 patients were considered for further screening. The full texts of the studies were extracted and subjected to inclusion criteria; systematic reviews and/or meta-analysis with extractable quantitative data on specified study outcomes. Narrative reviews, animal studies, and those published in languages other than English were excluded. The bibliography of eligible SRs was also checked for potentially eligible studies. The gray literature was searched using Google scholar and the search was continued up to 10 pages, or until the search does not reveal any article not captured by previous searches.

### Methodological appraisal using assessing the methodological quality of systematic reviews

The studies fulfilling the inclusion criteria were subjected to the quality assessment by using the assessing the methodological quality of systematic reviews (AMSTAR-2) tool. This tool evaluates the quality of SRs through 16 domains on various aspects; appropriateness of study selection, risks of bias (RoB) in primary studies, sensitivity analyses, the correctness of methods used for meta-analyses, consideration of RoB and heterogeneity while interpreting or discussing the results, and protocol registration. An online web-supported estimation was used to classify the studies into four quality categories; high, moderate, low, and critically low (9). The quality assessment of the studies was performed by two reviewers independently, where disagreement among authors was resolved through discussion and consensus by considering the third opinion from the principal investigator. Moreover, the quality of the included SRs was also assessed visually by tabulating each SR with information on the type of study, search strategies used, inclusion and exclusion criteria, methods used to evaluate RoB in primary studies, registration of SR, and major comments on RoB and heterogeneity in studies.

### Data extraction and reporting

Two reviewers (YHK, MHB) extracted the required data independently using a standardized data collection form. Any conflict or deviation was solved through mutual consultation and concurrence with final approval from another reviewer (THM). Data were included according to the Cochrane recommendations for an overview of reviews. The following data were extracted: authors, year, type of review, the total number of primary studies, the total number of participants, objectives of the review, name, and the number of databases searched, the methodological quality of studies, limitations in SRs, the pooled prevalence of CAKI, risk factors of development of AKI during COVID-19, need for RRT among COVID-19 patients, an association of CAKI with mortality, and other relevant information on CAKI. In the case of missing information in SR, a gap in data reporting was described as *not reported*. Since the primary objective of this overview was to summarize the current body of available evidence on CAKI, an overlap analysis of primary studies was not performed. According to the Cochrane handbook for systematic reviews, it is acceptable to include the results of all relevant SRs regardless of overlapping of primary studies if the overview intends to describe the available evidence on a specified topic (10).

### Strategies for data synthesis

**FIGURE 1:**
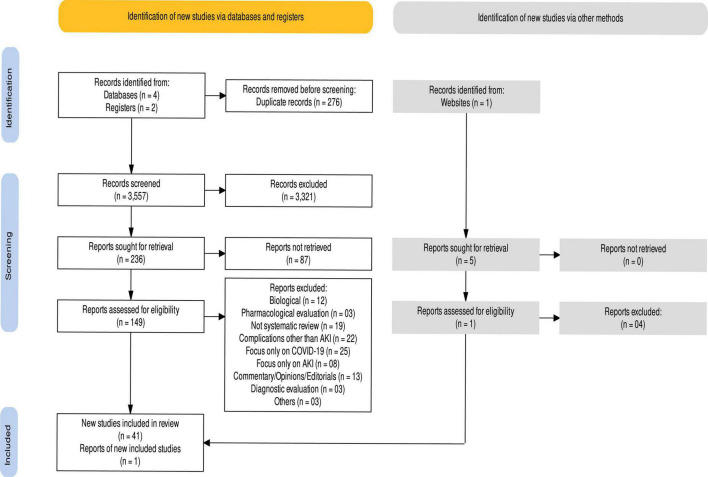
PRISMA flow diagram.

The extracted data were qualitatively synthesized and tabulated according to the aims of the study. Qualitative analysis of the findings from SRs was performed using narrative synthesis. Narrative summaries of the outcome data were presented within corresponding tables. The resulting data were also grouped according to study population i.e., adults, children, or kidney transplant recipients (KTRs). The pooled data from meta-analyses were presented and heterogeneity across the analyses was recorded as a minimum to maximum value for *I*^2^. The PRISMA flow diagram of this SR is shown in Figure 1. In addition, the PRISMA checklist has been provided in Supplementary Table 1.

### Operational definitions

This review has the following terms that need to be defined; COVID-19: confirmed COVID-19 cases, CAKI: acute kidney injury among COVID-19 patients, CAKI associated mortality: mortality rate among COVID-19 patients who developed AKI, Need for RRT: use of any modality of RRT among COVID-19 patients, risk factors of CAKI: factors or variables found to be associated with the development of AKI among COVID-19 patients. This review used various interchangeable terms which were originally used in included SRs e.g., RRT, CRRT, or dialysis are replaceable terms but they were used in this review as written in original SRs, as the definition of each term varied in SRs.

## Results

Initial search retrieved 3,833 articles from various databases and registers, where 276 were duplicates. Most of the SRs were excluded following abstract and title screening (*n* = 3,321) and 236 studies were subjected to full text screening. A total 42 reviews are included in this overview, where remaining studies were excluded due to the reasons described in Figure 1.

### Characteristics of included reviews

A total of 42 reviews (38 SRs with MA and 3 without MA) were included in this study (1, 2, 4, 11–49). The number of primary studies in the reviews ranged from 4 to 142 with confirmed COVID-19 cases ranging from *n* = 420 to *n* = 54,173. The primary studies in SRs were from various regions around the world, thereby covering all the global continents. None of the reviews included clinical trials (as they were not available on this topic), and three studies were published as research letters (12, 13, 27). Most of the reviews (*n* = 32, 76.2%) were reported according to PRISMA, MOOSE, and Cochrane checklists. However, nine studies (21.4%) did not include any information on reporting guidelines. The risk of bias (RoB) among primary studies was performed in 34 reviews (81%), while 8 studies (19%) did not appraise the primary studies. The New-Castel Ottawa Scale (NOS) was the most commonly (*n* = 14) used RoB tool in these reviews. Only eleven review protocols (26.2%) were registered before the synthesis of results. Six reviews (12, 16, 22, 24, 26, 27) which were published in 2020 did not provide details on specific inclusion and exclusion criteria. Most of the reviews excluded studies with < 10 patients, published in languages other than English, and on specialized populations i.e., children, pregnant women, and patients with cancer and CKD. One review was conducted on the pediatrics population (44), while five studies included KTRs in data synthesis (21, 31, 34, 36, 37). All the reviews did not include animal studies, and most of the reviews excluded case reports, except five studies (1, 11, 13, 21, 45). The heterogeneity across the primary studies was also observed by using *I*^2^-values from forest plots. Most of the reviews reported high heterogeneity with *I*^2^ > 90% during the analysis and sensitivity analysis was not considered in these studies. The methodological summary of included reviews is described in Table 2.

**TABLE 2:** Methodological summary of included reviews with reported heterogeneity across meta-analyses.

References	Type of review	Number of studies (total patients)	Primary objectives of the review	Reporting guidelines	Inclusion and exclusion criteria of the review	Quality assessment tools for primary studies	Heterogeneity among studies (for SR and MA) and risk of bias (for SR only)	Protocol registered
Ali et al. (11)	SR and MA	6 (*n* = 1,250)	Evaluation of severe AKI in COVID-19 patients	PRISMA, Cochrane and MOOSE	Included studies: All studies performed on human having baseline creatinine, occurrence of AKI stage III and/or need for acute RRT Excluded studies: Reviews, case reports	NOS	High (*I*^2^ > 95%)	No
Chen et al. (12)	SR and MA (Research letter)	20 (*n* = 6,945)	Incidence of CAKI	NR	Included studies: Studies reporting the AKI during the course of COVID-19 (no types of studies are mentioned) Excluded studies: No specific criteria were defined for study exclusion	NR	High (*I*^2^ = 97.8%)	No
Chen et al. (13)	SR and MA (Research letter)	23 (3 SARS, 4 MERS, 16 COVID-19), *n* = 11,323 (COVID-19 patients: 10,339)	Mortality comparison in patients with SARS, MERS, and COVID-19 who developed AKI	NR	Included studies: Cohort studies, case series, case reports, cross-sectional studies Excluded studies: Reviews, conferences proceedings, studies without report on AKI mortality	NR	High (*I*^2^ = 97%)	No
Cheruiyot et al. (14)	SR and MA	15 (*n* = 5,832)	Prognosis of CAKI and its association with disease severity and mortality, mortality in patients with severe disease	PRISMA	Included studies: Observational cohort and case control studies, studies with clear definition of COVID-19, severe disease, AKI (KDIGO criteria) Excluded studies: reviewers, studies having incomplete data	MINORS	Low (*I*^2^ = 0–90%)	Yes
Fabrizi et al. (15)	SR and MA	39, (*n* = 25,566)	Incidence of CAKI, requirement of CRRT, incidence of AKI in severe patients, impact of CAKI on death risk	PRISMA	Included studies: all types of studies on COVID-19 patients Excluded studies: Studies on pregnant women, without information between severe and non-severe patients, letters, case or interim reports, reviews	NR	High (*I*^2^ = 97.26%)	No
Figliozzi et al. (16)	SR and MA	49 (*n* = 20,211)	Predictors of adverse prognosis or clinical outcomes among COVID-19 patients	PRISMA	Included studies: Peer reviewed studies regardless of the language Excluded studies: No specific exclusion criteria was defined in the study	NOS	High (*I*^2^ = 93.8–95%)	Yes
Fu et al. (17)	SR and MA	142 (*n* = 49,048)	Incidence, outcomes, risk factors and need for KRT among COVID-19 patients	PRISMA	Included studies: Studies on more than 10 patients, 80% or more patients had age ≥ 18 years, cohorts, case controls, case series, clinical trials Excluded studies: cross-sectional studies, case reports, reviews, preprints, non-English	COSMOS-E	High (*I*^2^ = 89–98.7%)	No
Hansrivijit et al. (18)	SR and MA	26, (*n* = 5,497)	Incidence of AKI and the association between AKI and mortality in patients with COVID-19	PRISMA	Included studies: Observational studies Excluded studies: Reviews, case reports, studies on children	ROBINS-I	Moderate (*I*^2^ = 48% to)	No
Kunutsor and Laukkanen (19)	SR and MA	22, (*n* = 17,391)	Prevalence of renal complications in COVID-19 and association of pre-existing renal conditions with the occurrence of renal complications	PRISMA and MOOSE	Included studies: Observational studies, randomized and non-randomized clinical studies, studies with COVID-19 patients having follow-up prior to developing complications Excluded studies: Reviews, studies reporting no renal outcomes	NOS	High (*I*^2^ > 95%)	Yes
Lim et al. (20)	SR and MA	15, (*n* = 3,615)	Exploring the relationship of AKI with composite outcomes (mortality, severe infection and need for ICU care)	MOOSE	Included studies: research articles (published or pre-prints) on adult population Excluded studies: Reviews, commentaries, letters, case reports, non-English studies	NOS	Moderate (*I*^2^ = 0–24%)	No
Lin et al. (1)	SR and MA	79 (*n* = 49,692)	Risk factors and Prognosis of CAKI	NR	Included studies: Retrospective analyses, cross-sectional studies, and case reports related to confirmed COVID-19 Excluded studies: lacking clear diagnosis of AKI and COVID-19, reviews, guidelines, comments, basic research	Cochrane risk-of- bias criteria	Moderate (*I*^2^ = 19–83%)	No
Marinaki et al. (21)	SR	63, (*n* = 420)	Outcomes of COVID-19 in kidney transplant recipients	PRISMA	Included studies: Case reports, case series, cohort studies, correspondences, Excluded studies: Reviews, conference proceedings	JBI	High	No
Nogueira et al. (22)	SR	18, (*n* = 5,341)	Incidence of CAKI	PRISM	Included studies: all types of studies evaluating CAKI Excluded studies: No specific exclusion criteria was described in the study	NR	NR	No
Potere et al. (23)	SR and MA	44, (*n* = 14,866)	Evaluation of outcomes COVID-19 and factors predicting worse prognosis	PRISMA	Included studies: Observational studies, RCTs reporting at least one primary outcome Excluded studies: Case reports, case series with less than 50 patients	MINORS	High (*I*^2^ = 92%)	No
Robbins-Juarez et al. (4)	SR and MA	30, (*n* = 21,591) After removing overlapping cohorts 20, (*n* = 13,137)	Assessment of incidence of CAKI and its association with outcomes	PRISMA	Included studies: retrospective and prospective cohort or case series with > 20 participants Excluded studies: Studies on special population i.e., pediatrics, pregnant, transplant, ESKD, and cancer, reviews, cross sectional studies, RCTs on drug therapy, overlapped studies (only 1 study is included from studies having duplicate cohorts)	NHLBI Quality Assessment Tool for case series studies	High (*I*^2^ = 31.39–98.49%)	No
Shao et al. (24)	SR and MA	40, (*n* = 24,527)	Correlation between CAKI, disease severity and fatality	PRISMA and MOOSE	Included studies: Studies on COVID-19 patients reporting AKI Excluded studies: No specific exclusion criteria was reported	NOS	High (*I*^2^ = 77–98%)	No
Vakili et al. (25)	SR and MA	30, (*n* = 6,389)	Prevalence of critical complications in COVID-19	PRISMA	Included studies: All types of studies on various complications of COVID-19 Excluded studies: Reviews, animal and non-English studies	NR	High (*I*^2^∼95%)	No
Yang et al. (26)	SR and MA	24, (*n* = 4,963)	Prevalence of CAKI, risk of AKI in severe disease and non-survivors	NR	Included studies: No specific inclusion criteria was mentioned Excluded studies: Reviews, case reports, studies with < 10 patients, having only pediatric population, family-based studies, only written in Chinese language	NR	Moderate (*I*^2^ = 52–93%)	No
Zhang et al. (27)	SR and MA (Letter to editor)	4, (*n* = 789)	Evaluation of clinical characteristics and prognostic factors among patients with CAKI	NR	Included studies: No specific inclusion criteria was mentioned Excluded studies: Reviews and case reports	MINORS	Low (*I*^2^ = 0–73%)	No
Alenezi et al. (28)	SR and MA	31, (*n* = 27,500)	Evaluation of incidence and risk factors of CAKI among patients with and without ARDS	PRISMA	Included studies: observational cohort, cross-sectional studies, and case series having required data Excluded studies: reviews, case reports, case series with < 10 patients, Studies with pediatric, cancer, pregnant women, kidney transplant and ESRD patients	NHLBI quality assessment tool	High (*I*^2^ = 0–95%)	Yes
Brienza et al. (29)	SR and MA	10, (*n* = 5,166)	Assessment of incidence of CAKI and its relationship with mortality	PRISMA and MOOSE	Included studies: All types of studies on adult population Excluded studies: Studies conducted on pediatric population	Cochrane risk-of-bias criteria	Moderate (*I*^2^ = 29%)	No
Cai et al. (2)	SR and MA	38 (*n* = 42,779)	Risk factors of CAKI	NR	Studies on confirmed COVID-19 patients with age > 16 years Study designs: horizontal cross-sectional studies, case-control studies, and cohort studies	NOS	High (*I*^2^ = 16–96.6%)	No
Cau et al. (30)	SR and MA	COVID-19 studies: 71, (*n* = 54,173) ACE2-associated viruses’ study: 20, (*n* = 1,575) Non-ACE2 associated viruses’ studies: 3, (n = 370)	Prevalence of AKI and CRRT in critically ill COVID-19 patients (C) and its comparison with critically ill patients infected with ACE2-associated viruses (A) and non-ACE2 associated viruses (non-A) (Three groups: C, A, non-A)	NR	Included studies: cohort or case control studies, letters, commentaries, Systematic reviews and meta-analyses, Studies defining AKI with KDIGO definition Excluded studies: Studies with < 10 patients, cohorts of deceased patients and patients on dialysis or kidney transplant recipients, non-English	Risk of bias of studies were performed by un-identified questionnaire having 10 items	High for COVID-19 studies (*I*^2^ = 98.1%)	No
Chan et al. (31)	SR and MA	74, (*n* = 18,569, for AKI incidence analysis, total number of patients from 74 studies are not reported)	Evaluation of renal involvement in COVID-19 and its association with mortality	NR	Included studies: All types of observational studies regardless of language Excluded studies: Case reports, qualitative studies	NHLBI quality assessment tool	High (*I*^2^ = 63–99%)	Yes
Chan et al. (32)	SR and MA	25, (*n* = 4,032)	Assessment of urological manifestations	PRISMA	Included studies: Case reports, case series, observational studies, non-randomized studies and randomized trials Excluded studies: Letters, commentaries, studies on pregnant patients	NR	High (*I*^2^ = 61–96%)	No
Chang et al. (33)	SR and MA	59, (*n* = 5,956)	Analysis of outcomes of COVID-19 in KTRs, including mortality rate, acute kidney injury rate, invasive ventilation rate and rate of graft loss.	PRISMA	Included studies: Studies on COVID-19 patients admitted to ICU Excluded studies: Reviews, Case reports, abstracts, studies without outcomes of interest	NHLBI quality assessment tool for observational cohorts	High (*I*^2^ > 80%)	Yes
Chen et al. (34)	SR and MA	28, (*n* = 12,437)	Incidence of Mortality, AKI and Graft Loss in adult KTRs with COVID-19	PRISMA	Included studies: Studies enrolled adult kidney transplant patients having COVID-19 Excluded studies: Reviews, conferences proceedings, studies without report on AKI mortality	NOS	High (*I*^2^ = 57–83%)	Yes
Daniella et al. (35)	SR	4, (*n* = 6,051)	Risk factors of CAKI	PRISMA	Included studies: studies having diagnosis of COVID-19 with RT-PCR, AKI with KDIGO criteria, and AKI occurs during hospitalization, evaluating the risk factors of CAKI with odds ratio Excluded studies: non-English, reviews, animal studies	ROBINS-I	Moderately high risk of bias	No
Ho et al. (36)	SR and MA	23, (*n* = 1,373)	Examining clinic-laboratory features and outcomes among KTRs with COVID-19	PRISMA	Included studies: Authors did not specify any inclusion criteria Excluded studies: Studies with < 5 patients	JBI critical appraisal checklist	Low (*I*^2^ = 9–20%)	Yes
Kremer et al. (37)	SR and MA	74, (*n* = 5,559)	Clinical Course and outcomes of COVID-19 in KTRs	PRISMA	Included studies: All types of studies (even research posters) with at least five kidney transplant recipients Excluded studies: Animal studies, non-English	NOS	High (*I*^2^ = 66–83%)	Yes
Lee et al. (38)	SR and MA	14, (*n* = 17,876)	Association between RAAS blockade use and AKI development in COVID-19 patients.	PRISMA	Included studies: case–control, retrospective or prospective cohort, or descriptive studies, studies must have data on the use of RAAS blockade Excluded studies: Reviews, case reports, case series with < 10 patients, non-English studies	NOS	Moderate (*I*^2^ = 80–89%)	No
Liu et al. (39)	SR and MA	36, (*n* = 6,395)	Is CKD a risk factor of severe COVID-19, is AKI a complication for severe COVID-19?	PRISMA	Included studies: Case series, cohorts, and prospective studies among adults having data on COVID-19 association with kidney Excluded studies: Reviews, studies on patients who were already receiving RRT	NOS	Low (*I*^2^ < 50%)	No
Menon et al. (40)	SR and MA	20, (*n* = 14,415)	Incidence of CAKI, outcomes related to disease severity, prognosis of CAKI	PRISMA	Included studies: Prospective and retrospective, cohort studies having COVID-19 patients with extractable data of AKI Excluded studies: reviews, studies on CKD, ESR, cancer and pediatric patients	NOS	High (*I*^2^ = 98%)	No
Oliveira et al. (41)	SR and MA	21 (*n* = 15,536)	Evaluation Incidence of AKI in COVID-19, incidence of CAKI in critically ill cases, RRT and mortality in CAKI, risks of death in AKI vs. non-AKI	PRISMA and MOOSE	Included studies: studies having COVID-19 diagnostic methods, AKI identification and classification Excluded studies: studies on pregnant, ESRD and transplant patients, studies not using KDIGO criteria for AKI, case reports, non-English	NHLBI quality assessment tool	High (*I*^2^ = 67–98%)	No
Ouyang et al. (42)	SR and MA	41, (*n* = 10,335)	Association of AKI with the severity and mortality of SARS-CoV-2	PRISMA	Included studies: Studies on adults reporting renal impairments Excluded studies: Reviews, case reports, studies on special population (children, elderly, pregnant women, transplant recipients and cancer patients), studies having < 10 patients	NOS	Low (*I*^2^ = 0%)	No
Passoni et al. (43)	SR and MA	30, (*n* = 18,043)	Incidence of CAKI	PRISMA	Included studies: Studies with required data and having patients with age > 18 years, published in English, Spanish and Portuguese Excluded studies: studies on children and adolescences, with < 10 patients, studies on CKD and kidney transplant patients	JBI Critical appraisal tool	High (*I*^2^ > 95%)	Yes
Raina et al. (44)	SR and MA	60, (*n* = 42,612)	Incidence and outcomes of CAKI	PRISMA	Included studies: Prospective and retrospective cohort studies, case series among adults and pediatrics Excluded studies: reviews, case reports, RCTs, animal studies, Studies exclusively on kidney transplant patients, non-English	NR	High (*I*^2^ > 95%)	No
Raina et al. (45)	SR and MA	24, (*n* = 1,247 (from 14 studies with sample size ≥ 10)	Incidence of AKI, associated mortality and need for KRT, and outcomes in pediatric COVID-19 population	PRISMA	Included studies: Prospective and retrospective, case reports, case series on pediatric population evaluating AKI in COVID-19 Excluded studies: Reviews, animals’ studies, with population > 24 years old	NHLBI quality assessment tool	High (*I*^2^∼90%)	No
Silver et al. (46)	SR and MA	54, (*n* = 30,657)	Evaluation of Prevalence of AKI and KRT in COVID-19 patients	PRISMA and MOOSE	Included studies: Studies reporting prevalence of AKI according to KDIGO criteria Excluded studies: studies having < 20 patients, patients with age less than 18 years old, missing data, non-English, reviewers, letters, commentaries,	NHLBI quality assessment tool for case series	High (*I*^2^ = 76–99%)	No
Xu et al. (47)	SR and MA	22, (*n* = 16,199)	Incidence of CKI, incidence of CAKI in remdesivir treated patients	NR	Included studies: Observational studies among COVID-19 patients being treated with remdesivir Excluded studies: Reviews and case reports, Studies not using KDIGO criteria for AKI	AHRQ methods for retrospective cross-sectional studies and Cochrane Collaboration tool for RCTs	High (*I*^2^∼90% in AKI)	No
Yang et al. (48)	SR and MA	58, (*n* = 22,671)	Incidence of AKI and RRT among COVID-19 patients	PRISMA	Included studies: Observational studies reporting AKI and RRT rates in COVID-19 Excluded studies: Reviews, letters, and case report, non-peer reviewed studies	NOS	High (*I*^2^ = 67–98%)	No
Zhou et al. (49)	SR and MA	58, (*n* = 13,452)	Assessment of renal involvement in patients with COVID-19, SARS and MERS	PRISMA	Included studies: Case series, cohorts, and case control studies having required data Excluded studies: Studies having < 5 patients	NOS	High (*I*^2^ = 83–98%)	Yes

ACE2, Angiotensin-Converting Enzyme 2; AHRQ, Agency for Healthcare Research and Quality; AKI, Acute kidney injury; CAKI, COVID-19 induced AKI; CKD, Chronic Kidney Disease; COSMOS-E, Conducting Systematic Reviews and Meta-Analyses of Observational Studies of Etiology; CRRT, Continuous renal replacement therapy; ESKD, End stage kidney disease; ESRD, End stage renal disease; JBI, Joanna Briggs Institute’s () critical appraisal checklist; KRT, Kidney replacement therapy; KTR, Kidney transplantation recipients; MA, meta-analysis; MERS, Middle East Respiratory Syndrome; MINORS, Methodological index for non-randomized studies tool; MOOSE, Meta-analyses of Observational Studies; NHLBI, National Heart, Lung, and Blood Institute; NOS, Newcastle–Ottawa scale; PRISMA, Preferred Reporting Items for Systematic Reviews and Meta-Analyses; RCTs, Randomized Controlled Trials; ROBINS-I, Risk of Bias in Non-randomized Studies of Interventions tool by Cochrane; RRT, Renal replacement therapy; SARS, Severe acute respiratory syndrome; SR, systematic review.

### Search databases used in included reviews

**FIGURE 2:**
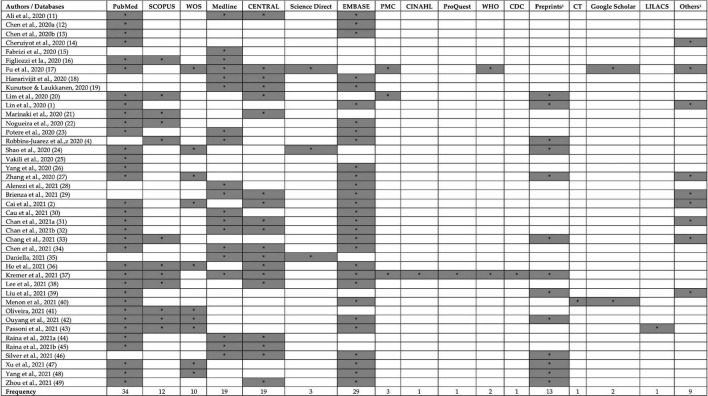
Search databases used in included reviews. CDC, Centers for Disease Control and Prevention database; CENTRAL, Cochrane Central Register of Controlled Trials; CINAHL, Cumulated Index to Nursing and Allied Health Literature; CT, ClinicalTrials.gov; PMC, PubMed Central; WHO, world health organization database; WOS, Web of Science. ^1^Preprints: BioRvix, MedRvix, and ^2^Other databases: Chinese databases, Intensive Care National Audit and Research Center (ICNARC) website, DARE database, CNKI, VIP, WanFang. The symbol * represents the presence database.

PubMed (*n* = 34), EMBASE (*n* = 29), Medline (*n* = 19), CENTRAL (*n* = 19), Preprints (*n* = 13), and SCOPUS (*n* = 12) were the most commonly used search databases in included SRs. Most of the studies used ≥ 2 databases with an average of 3.8 databases per review. However, two reviews only searched one database to retrieve the relevant studies (15, 25). Preprints and Chinese databases were also searched in various reviews (Figure 2).

### Methodological appraisal using assessing the methodological quality of systematic reviews

**FIGURE 3:**
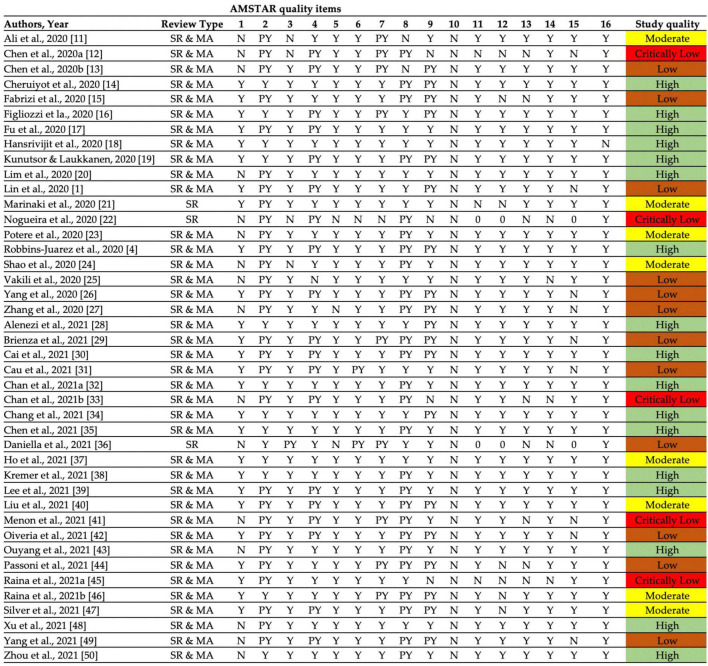
Quality assessment of included systematic reviews and meta-analyses according to AMSTAR-2 checklist. (High: Zero or one non-critical weakness, Moderate: More than one non-critical weakness, Low: One critical flaw with or without non-critical weaknesses, Critically low: More than one critical flaw with or without non-critical weaknesses).

Approximately half of the reviews included (*n* = 17, 40.5%) were qualitatively judged as low (Low: 12, Critically low: 5). Only 17 (40.5%) reviews were judged as of high quality, while 8 (19.0%) studies were of moderate quality. None of the reviews provided the funding information for the primary studies (Figure 3). The quality appraisal indicates that most studies have critical flaws and may not provide accurate evidence synthesis. In this context, the major results were combined from studies having moderate to high methodological quality according to the AMSTAR-2 criteria. Seventeen reviews were excluded from the current overview due to poor methodological quality as reporting from low-quality reviews hinders concluding results for better understanding. However, all low-quality results were presented in Supplementary Table 2.

### Incidence of COVID-19 associated acute kidney injury

Nineteen reviews reported the pooled incidence of AKI either in overall COVID-19 patients or in special populations (Table 3). The overall incidence of CAKI ranged from 4.3% (39) to 30.51% (45). However, the incidence of CAKI in specialized populations is given below;

#### COVID-19 positive kidney transplant recipients

Four reviews reported CAKI in KTRs ranging from 36% (31) to 50% (37). Fu et al. reported AKI in transplant patients ranging from 30% to 69%, but pooled prevalence was not estimated (17).

#### Patients with severe or critical COVID-19 infection

The definition of severe infection widely varied across the studies. Only one review reported pooled prevalence of CAKI as 21.1% in severe infection (39). However, six reviews (17, 18, 33, 34, 46, 47) pooled the incidence of CAKI from 19.9% (18) to 46% (46) among critically ill patients.

#### Patients with non-severe, non-critical or mild to moderate COVID-19 infection

Only one study provided the estimation of CAKI as 12% among COVID-19 patients who were not admitted to ICU. None of the included SR reported the pooled prevalence of CAKI in non-severe, mild or moderate cases of infection. Such prevalence was pooled in two low quality reviews (1, 22). One SR indicated that AKI occurred in 59% of patients with ARDS and 6% among patients without ARDS (28).

#### Prevalence of acute kidney injury stages

Two SRs (4, 46, 48) pooled the incidence of AKI stages; stage I: 15%–44%, stage II: 7%–19%, and stage III: 11%–34%. AKI stage I was most prevalent across the SRs included in this review.

#### Pediatric and elderly population

One SR reported the pooled prevalence of CAKI among children as 16.1% (31). Fu et al. reported pooled prevalence of CAKI as 12% in patients with age > 60 years, while it was 6% among patients having age < 60 years (17).

#### COVID-19 deceased patients

One SR reported CAKI as of 30.72% (42) among deceased patients. The odds (OR) of AKI among deceased patients was reported as 77.48 (42) in the same review.

#### COVID-19 patients from various geographical regions

Some reviews stratified the analysis according to the geographical location of the primary studies. Two reviews reported the incidence of CAKI ranging from 8.2% (19) to 9% in China. Two reviews reported CAKI ranging from 5.5% (17) to 7% (47) in Asia. One SR indicated CAKI as 28.6% in the USA and Europe (17). The reported incidence in the USA alone was 19.9% (19).

#### Miscellaneous patient groups

Xu et al. reported 7% AKI among COVID-19 who were receiving remdesivir therapy (47). Many low-quality studies have reported pooled prevalence of CAKI among patients with early hospitalization, secondary infection, sepsis, septic shock and those infected with ACE2-associated viruses but the data was not presented here as these studies were of low quality.

### Risk factors of COVID-19 associated acute kidney injury

Only five SRs reported the risk factors for the development of AKI during COVID-19 infection. The most commonly reported risk factors were diabetes mellitus, hypertension (2, 17, 18, 31), chronic kidney disease (2, 17, 31), cardiovascular disease (2, 17, 31), age (2, 17, 18), and male gender (2, 17, 31). Other predictors were coronary artery disease (31), smoking, obesity, cancer, pneumopathy, mechanical ventilation, vasopressor use (2), and blockade of renin-angiotensin aldosterone system (RAAS) (38). Most of these factors were reported through meta-regression analyses with odd ratios (Table 3).

### Need for renal replacement therapy

Sixteen reviews reported the need for RRT among COVID-19 patients. However, the modalities of RRT were not adequately described in these reviews. The RRT was needed by 1% (28)–23% (21) of COVID-19 patients. However, the need for RRT was reported as 15.6% in one review (47). One review reported that 12.65% of kidney transplant patients needed RRT (31). Two reviews (17, 46) reported the need for RRT ranging from 19% (46) to 26.6% (17) among critically ill patients, while its need was only 1% in non-critical patients (46). One review (39) showed RRT needs among patients with severe infection, having an odds ratio of 23.63 (39). Chan et al. reported 5.54% of children needed RRT during the COVID-19 treatment (31). However, the RRT was needed in 31.51% of fatal cases (42). The pooled incidence of RRT was found to be comparatively high among COVID-19 patients with ARDS (20%) than those without ARDS (1%) (28) (Table 3).

### Mortality associated with COVID-19 associated acute kidney injury

**FIGURE 4:**
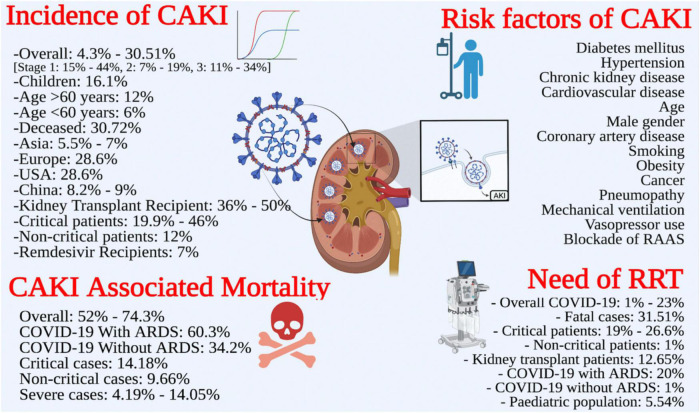
Review summary: incidence, risk factors, need for renal replacement therapy (RRT), outcomes of CAKI.

Fourteen reviews reported the association of CAKI with increased mortality (Table 3). The CAKI-associated mortality rate ranged from 52% (4) to 74.3% (49). In addition, the prevalence of mortality with the urgent start of RRT was 74.2%, having an odds ratio of 3.04. Ten reviews (4, 11, 14, 16, 18, 24, 28, 31, 34, 45, 49) found CAKI to be an independent risk factor of mortality with odd ratios ranging from 2.55 (45) to 23.9 (14). However, the odds of mortality due to CAKI in severe COVID-19 infection ranged from 4.19 (11) to 14.05 (14). One review reported the relationship of AKI severity with mortality, where AKI stages 1, 2, and 3 had 7.45, 24.64, and 94.77 times higher odds of death as compared to patients who did not develop AKI (31) (Table 3). The critical cases portended higher mortality than non-critical cases (14.18% vs. 9.66%) (4). Likewise, COVID-19 patients with ARDs had higher mortality as compared to those without ARDS (60.3% vs. 34.2%) (28) (Figure 4).

**TABLE 3:** Major findings extracted from the moderate and high-quality reviews (incidence, risk factors, need of RRT and outcomes of CAKI).

References	Pooled prevalence/ Incidence of CAKI	Risk factors of CAKI (OR/MD/Q/WMD/SMD)	Need for RRT	CAKI associated mortality or severe disease or other adverse outcomes	Comments
Ali et al. (11)	NR	NR	NR	Odds of severe CAKI for mortality (OR): 4.19 (2 studies)	Patients with CAKI have higher mortality even after initiation of RRT
Cheruiyot et al. (14)	NR	NR	NR	Odds of mortality due to CAKI (OR): 23.9 (6 studies) Odds of mortality among patients with severe COVID-19 infection and CAKI (OR): 14.05 (2 studies) Odds of Severe COVID-19 due to CAKI (OR): 18.5 (8 studies)	This review has presentation error as forest plot for mortality and severe disease due to CAKI look similar to each other.
Figliozzi et la. (16)	NR	NR	NR	Odds of composite adverse outcomes for CAKI: 5.3% Odds of mortality due to CAKI: 12.1 (14 studies)	The presence of AKI in COVID-19 is associated with adverse outcomes along with death.
Fu et al. (17)	Overall CAKI: Asia = 5.5% (62 studies) Europe/USA = 28.6% (20 studies) Kidney Transplant patients: 30–69% (5 studies) ICU patients: 29.2% (23 studies)	Odds ratio per 10 years or 10% increase Age: 2.15 (83 studies) Male: 1.36 (83 studies) Cardiovascular disease: 1.53 (64 studies) CKD: 1.64 (83 studies) Diabetes: 1.48 (72 studies) Hypertension: 1.50 (72 studies)	Need for KRT: China = 2.2% (52 studies) EU/USA = 7.7% (18 studies) Need for KRT in kidney transplant patients: 0–21% (5 studies) Need for KRT in ICU patients: 26.6% (38 studies)	Pooled risk ratio (RR) of mortality = 4.6	Death and hospital discharge in > 80% patients occurred in 39% of studies The heterogeneity among the studies for which Odds ratio was estimated was > 90% Risk factors and outcomes were not adjusted for confounders in this review
Hansrivijit et al. (18)	Overall CAKI: 8.4% (26 studies) Critically ill patients: 19.9% (5 studies)	Meta-regression adjusted for gender and CKD: Age: *Q* = 10.79, *p* = 0.02 DM: *Q* = 19.16, *p* < 0.01 HTN: *Q* = 11.65, *p* = 0.02 Baseline SCr: *Q* = 15.43, *p* < 0.01	Need for RRT: 3.6% (14 studies)	Odds of mortality due to CAKI: 13.33 (8 studies)	The further adjustment of meta-regression model for age, comorbidities and serum creatinine levels showed that only baseline SCr is associated with AKI
Kunutsor and Laukkanen (19)	Overall CAKI: 11% (22 studies) China: 8.2% USA: 19.9%	NR	Need for RRT: 6.8% (3 studies)	NR	Electrolyte disturbances (particularly hyperkaliemia) are also common in COVID-19. The studies with high prevalence of pre-existing CKD among showed higher incidence of AKI (31.8%)
Lim et al. (20)	NR	NR	NR	Risk ratio of Composite outcomes with AKI (RR): 10.55 Risk ratio of mortality due to CAKI (RR): 13.38 (7 studies) Risk ratio of severe COVID-19 with CAKI (RR): 8.12 (6 studies) Risk ratio of need of ICU care with AKI (RR): 5.90	Many studies in this review were pre-prints, and were from China. Overlapping patients were not ruled out The association of AKI with composite endpoint was not influenced by age, gender and other co-morbidities
Marinaki et al. (21)	Overall CAKI: 44%	NR	Need of RRT in CAKI: 23%	Prevalence of CAKI in fatal cases: 58%	The most of the studies were case reports. COVID-19 patients who received antithymocyte globulin had lower prevalence of AKI (38%)
Potere et al. (23)	Overall CAKI: 6% (15 studies)	NR	NR	NR	Very low quality evidence due to heterogeneity and risk of bias, overall mortality was 10% (43 studies)
Robbins-Juarez et al. (4)	Overall CAKI: 17% (0.5 to 80.3%) (20 studies) Pooled prevalence (6 studies): Stage 1: 15% Stage 2: 7% Stage 3: 11%	NR	Need for RRT: 5% (8 studies)	Mortality among patients with CAKI: 52% (range: 7–100%) (9 studies) Odds of mortality due to CAKI (OR): 15.27 (9 studies) Odds of mortality due to CAKI (OR): 6.2 (6 studies, after excluding 3 studies with very high mortality in sensitivity analysis) Odds of mortality in Studies with ≥ 50% severe/ICU CAKI patients (OR): 14.18 Odds of mortality in studies with < 50% severe/ICU admitted CAKI patients (OR): 9.66 (2 studies)	Authors excluded cross sectional study from the analysis It was not clear that RRT was used solely for AKI or for some other conditions. A total of 77% patients with CAKI had either severe infection or ICU admission. Regional analysis on COVID-19 associated mortality showed higher odds of CAKI associated mortality in China (OR: 39, 5 studies) followed by USA (OR: 9.66, 2 studies) and Europe (OR: 3.56, 2 studies)
Shao et al. (24)	Overall CAKI: 10% (40 studies) Subgroups: China: 9% (30 studies) Internationally: 15% (10 studies) Odds of CAKI in severe infection (OR): 8.11 (24 studies)	NR	NR	Prevalence of mortality in COVID-19: 20.3% (20 studies) Prevalence of mortality in CAKI: 63.1% (20 studies) Odds of CAKI for mortality (OR): 14.63 (20 studies)	The high levels of SCr and BUN was associated with high fatality and severe disease The findings are biased by confounders Overlapped patients were not addressed in this review
Alenezi et al. (28)	Overall CAKI: 26% CAKI in Non-ARDS patients: 6% (3 studies) CAKI in ARDS patients: 59% (5 studies)	NR	Need for KRT: Overall patients: 6% (25 studies) Non-ARDS patients: 1% (3 studies) ARDS patients: 20% (4 studies)	CAKI associated mortality in patients with ARDS: 60.3% (8 studies) CAKI associated mortality in patients without ARDS: 34.2% (8 studies) Risk rate of mortality due to CAKI: 4.46 (16 studies)	The CAKI was more common in patients admitted to ICU or having ARDS
Cai et al. (2)	NR	Male (OR): 1.37 (36 studies) Older age (MD): 5.63 (32 studies) Smoking (OR): 1.23 (10 studies) Obesity (OR): 1.12 (10 studies) Hypertension (OR): 1.85 (31 studies) Diabetes (OR): 1.71 (37 studies) Pneumopathy (OR): 1.36 (27 studies) CVD (OR): 1.98 (35 studies) Cancer (OR): 1.26, (23 studies) CKD (OR): 4.56, (24 studies) MV (OR): 8.61, (25 studies) Use of vasopressors (OR): 8.33 (15 studies)	NR	NR	The CVD risk factor was further classified into various groups, resulting in discrete odds ratio for CAKI i.e., coronary heart disease (OR: 1.77), heart failure (OR: 2.41), and other CVDs (OR: 1.72)
Chan et al. (31)	Overall CAKI: 20.40% (17 studies) Patients with history of kidney transplantation: 35.99% Pediatric population: 16.11%	Categorical variables Male (OR): 1.22 (6 studies) DM (OR): 2.61 (6 studies) HTN (OR): 4.07 (6 studies) CKD (OR): 3.20 (3 studies) CAD (OR): 1.68 (3 studies) Continuous variables (difference AKI vs. non-AKI) *Old Age*: WMD: 8.39 years, SMD: 0.57 (6 studies) ↑ CRP at admission: WMD: 22.12 mg/L, SMD: 0.81 (4 studies) ↑ SCr at admission: WMD: 33.79 μmol/L, SMD: 0.75 (5 studies) ↓ Serum albumin at admission: WMD: -1.91 g/L, SMD: -0.37 (4 studies)	Need of RRT in COVID-19: 2.97% (17 studies) Need of RRT in pediatric COVID-19: 5.54% (2 studies) Need of RRT in kidney transplant patients: 12.65% (4 studies)	Mortality Odds of mortality due to CAKI (OR): 9.03 (17 studies) Odds of mortality in Stage 1 AKI (OR): 7.45 Odds of mortality in Stage 2 AKI (OR): 24.64 Odds of mortality in Stage 3 AKI (OR): 94.77 Critical disease Odds of being critical due to CAKI (OR): 17.58	Patients with kidney transplant history had a higher incidence of AKI but unexpectedly lower mortality when compared to those without kidney transplant history.
Chang et al. (33)	Overall CAKI: 44% (38 studies)	NR	Requirement of KRT among CAKI patients: 30% (27 studies)	Incidence of Mortality in transplant patients: 21% (58 studies) ICU admission rate in transplant patients: 26% (32 studies) Graft loss among transplant patients: 8% (11 studies)	The risk of mortality increased in proportion with the patients‘ age.
Chen et al. (34)	CAKI in ICU: 32% (6 studies)	NR	NR	Odds of mortality due to CAKI (OR): 12.47 (3 studies)	Most of the studies were pre-prints
Ho et al. (36)	Overall CAKI: 38.94% (17 studies)	NR	Need for dialysis: 12.37% (16 studies)	NR	KTRs with COVID-19 may have higher risks of developing AKI, requiring dialysis, and mortality
Kremer et al. (37)	Incidence of CAKI in KTR patients: 50% (42 studies) Incidence of CAKI in KTR patients with age < 50 years: 41% Incidence of CAKI in KTR patients with age > 60 years: 54	NR	NR	NR	Subgroup analysis showed that incidence of AKI was lower in Asia/Pacific region (39%) than USA (53%) and Europe (50%). Comorbidities were not associated with mortality and occurrence of COVID-19
Lee et al. (38)	NR	RAAS blockade: 1.68 (14 studies) ACEIs use: 2.32 (4 studies) ARBs use: 2.45 (5 studies)	NR	NR	RAAS blockade was significantly associated with moderate-to-severe AKI compared to no/mild AKI
Liu et al. (39)	Overall CAKI: 4.3% (8 studies) Severe COVID-19: 21.1% Odds of AKI in severe group (OR): 11.02 (8 studies) Odds of AKI in critical group (OR): 13.92 (3 studies)	NR	Odds of need for CRRT in severe group (OR): 23.63 (6 studies)	Severe vs. non-severe COVID-19 patients: Odds of CKD in severe group (OR): 3.28 (13 studies) Odds of abnormal serum creatinine in severe group (OR): 4.86 (7 studies) Odds of abnormal BUN (OR): 6.53 (4 studies) Critical vs. severe COVID-19 patients: Odds of CKD in critical group (OR): 1.3 (3 studies)—no significant difference	All studies were from China The statistical heterogeneity across the studies was not reported SCr did not differ between critical and severe group The critical group had higher ratio of AKI than severe group
Ouyang et al. (42)	Severe Cases: 26.74% Odds of AKI in severe cases: 13.16% (19 studies) Prevalence of CAKI in fatal cases: 30.72% (4 studies) Odds of CAKI in fatal cases: 77.48	NR	Odds of CRRT in fatal cases: 31.51	NR	Increased SCr, BUN, proteinuria and hematuria were more common in severe COVID-19. The impact of the drug-induced AKI cannot be ruled out in this study
Raina et al. (45)	Overall CAKI: 30.51% (14 studies)	NR	Prevalence of KRT in CAKI: 0.56% (4 studies)	Mortality rate in CAKI: 2.55% Incidence of multisystem inflammatory syndrome in children (MIS-C): 74.29%	Ten studies had sample size < 10, thus excluded (small sample size is a major barrier of this MA) Authors also included 7 case reports in MA
Silver et al. (46)	Overall CAKI = 28% (54 studies) ICU Patients: Prevalenc e of AKI = 46% (25 studies) Non-ICU patients Prevalence of AKI = 12% (9 studies) Pooled prevalence (13 studies): Stage 1: 44% Stage 2: 19% Stage 3: 34%	NR	Need of KRT: Overall Patients: 9% (48 studies) ICU patients: 19% (23 studies) Non-ICU patients: 1% (8 studies)	NR	One pre-print is also included, significant heterogeneity
Xu et al. (47)	Overall CAKI: 10% (22 studies) Asian ethnicity: 7% (14 studies) break Non-Asian: 15% (8 studies) Age ≥ 60 years: 12% (13 studies) Age < 60 years: 6% (9 studies) ICU: 26% (3 studies) Remdesivir treated patients: 7%	NR	Need of CRRT: Overall COVID-19: 4% (12 studies) Need for CRRT CAKI patients: 15.6%	NR	Since the use of antiviral drugs is linked with AKI. However, this study showed no association between the use of remdesivir with AKI, as 10% COVID-19 patients who were not treated with remdesivir also had AKI.
Zhou et al. (49)	Overall CAKI: 9%	NR	Rate of urgent-start KRT use: 3.4% (13 studies)	Odds of CAKI for mortality (OR): 5.22 (9 studies) Mortality rate in CAKI: 74.3% Odds of mortality in COVID-19 patients with urgent start of KRT (OR): 3.04 (6 studies) Mortality rate in COVID-19 patients with urgent start of KRT: 74.2% (6 studies)	The mortality was higher among COVID-19 patients with ESRD and pre-dialytic CKD patients. The incidence of AKI is similar between COVID-19 and SARS

ACE, angiotensin converting enzyme; ACEIs, angiotensin converting enzyme inhibitors; AKI, acute kidney injury; ARBs, angiotensin receptor blockers; ARDS, acute respiratory distress syndrome; BUN, blood Urea Nitrogen; CAD, Coronary artery disease; CAKI, COVID-19 associated AKI; CKD, chronic kidney disease; COPD, chronic obstructive pulmonary disease; COVID-19, Coronavirus Disease 19; CRRT, continuous renal replacement therapy; CVD, cardiovascular disease; DM, diabetes Mellitus; eGFR, estimated glomerular filtration rate; HF, Heart Failure; HPL, Hyperlipidemia; HTN, Hypertension; ICU, intensive care unit; KRT, kidney replacement therapy; KTRs, kidney Transplant recipients; MV, mechanical ventilation; NR, not reported; RAAS, Renin angiotensin aldosterone system; RR, risk ratio; RRT, renal replacement therapy; SARS, Severe acute respiratory syndrome; SCr, serum creatinine; SMD, standardized mean difference; WMD, weighted mean difference.

## Discussion

This is the first study of its own kind overviewing the existing data on CAKI. The large volume and variable quality of published work on COVID-19 highlight an overwhelming need to organize and summarize findings so that the most current and accurate information can easily be accessed. Though evidence in the form of SRs relating to the development of AKI during the COVID-19 infection exists but is conflicting. In this context, bringing together reviews transparently and systematically and aiding informed decision-making by gathering, appraising, and systematically analyzing the evidence have a valuable role during this ongoing pandemic. The findings originated from this SR generate pivotal insights and understanding of CAKI, thereby summarizing and enhancing the accessibility of existing evidence.

The kidney involvement during the COVID-19 infection ranges from mild proteinuria to an advanced AKI. The pathogenesis of SARS-CoV-2 mediated kidney injury is found to be complex and multifactorial. SARS-CoV-2 mainly binds with ACE2, which is expressed in kidneys on the brush border of the apical membrane of proximal tubules and to a lesser extent in podocytes. The receptor-binding domain (RBD) of the viral spike protein and transmembrane protease serine 2 (TMPRSS2) facilitates the entry of SARS-CoV-2 into renal cells (50). In addition, the upregulation of the NRP-1 receptor (due to arterial injury in severe COVID-19 patients) is also associated with viral invasion into the renal cells (51). Other mechanisms underlying the renal injury during the COVID-19 infection are cytokine storm, Angiotensin II pathway activation, dysregulation of complement, hypercoagulation, and microangiopathy (52, 53).

It is pertinent to mention that some of the currently approved drugs for the treatment of COVID-19 have nephrotoxic potential, their role in perpetuating renal damage cannot be disregarded. On the other hand, many drugs being used for the treatment of chronic ailments may also portend nephrotoxicity among COVID-19 patients. However, very few studies have investigated the impact of drugs on kidney functions among COVID-19 patients. Xu et al. quantitively synthesized the impact of remdesivir on kidney functions from five observational studies (*n* = 972 patients). The pooled incidence of AKI among patients who were using remdesivir was 7%, while it was 10% in patients who were not receiving remdesivir. Their findings indicate that remdesivir does not induce AKI among COVID-19 patients (47). Another study reported no association of remdesivir with drug-induced AKI even in patients who had a baseline eCrCl < 30 mL/min (54). In contrast, Lee et al. reported two times higher risks of developing moderate to severe AKI among ACEIs and ARBs‘ users compared to no/mild AKI. These findings underscored the relationship of RAAS blockade with the development of AKI among COVID-19 patients (38). The possible mechanism may relate to the significance of ACE2 during the pathogenesis of COVID-19. However, these hypothetical risks require more investigations to make a firm conclusion. The contribution of drugs in synergizing the renal damage among COVID-19 patients can only be assessed through large-scale studies. Likewise, the role of chronic diseases such as diabetes mellitus should also be considered while ascertaining the pathogenesis of kidney injury due to SARS-CoV-2.

The existing evidence indicates that the renal intricacies during COVID-19 infection are common and may require RRT. This study found a great disparity in the incidence of CAKI where its occurrence varied from 4.3% (39) to 30.51% (44) in overall COVID-19 patients. The wide variations in the incidence of CAKI across these SRs might be attributed to various factors i.e., disease severity, sample size, risk of bias in primary studies, demographic variations among patients (comorbidities, pre-existing renal impairment, use of nephrotoxic drugs), study design, diagnostic bias, inappropriate methods for statistical combination of results leading to under- or over-estimation of actual incidence, variation in methods used to estimate the baseline serum creatinine and variable operational definitions such as diagnostic criteria of AKI used in the primary studies. It is pertinent to mention that the reviews reporting the lowest (23, 26, 39) and highest (28, 35, 42, 45, 46) incidence of CAKI did not explain the reasons for such variations. However, the disease severity was reported as the most common contributor to the variable incidence of CAKI among COVID-19 patients. It can also be explained by the methodological quality status of the SR, as a low quality review reported the pooled incidence of CAKI among critically ill patients as 80.6% (41). The quality appraisal must be accounted for while interpreting the results from the reviews as poorly designed review methods may lead to erroneous results. Taken together, the findings of this overview indicate that CAKI is a commonly occurring atypical complication among COVID-19 patients. However, careful evaluation of these SRs indicated that various patient-related factors contributed to the higher incidence i.e., severe or critical illness, pre-existing renal anomalies, and renal transplants.

Interestingly, the incidence of AKI among COVID-19 patients was also compared with that reported in ACE2 (SARS-CoV-1, influenza H1N1, H7N9) and non-ACE2 (MERS-CoV, other influenza strains) viruses associated respiratory infections (30). The subgroup analysis showed that the frequency of AKI occurrence among COVID-19 patients (51%) was equivalent to that reported in infections associated with other ACE2 viruses (52%), while lower than that reported in non-ACE2 viruses associated infections (63%). Similarly, another study estimated the pooled prevalence of AKI in three coronavirus-related infections and found the highest prevalence of AKI in MERS (42%), followed by SARS (9.6%) and COVID-19 (9%) (49). Additionally, the need for RRT was also highest among MERS patients (30). It has been hypothesized that coronaviruses share common biological mechanisms to induce AKI among patients. However, the variation in the prevalence of AKI lends support to distinct pathophysiological processes among coronaviruses (30). For example, high renal expression of dipeptidyl peptidase-4 (receptor for MERS-CoV) might be attributed to the high prevalence of AKI and RRT need among MERS patients (55). Another study pooled the mortality rate due to AKI among coronavirus infections (SARS, MERS, COVID-19) at 77.4%, where the highest mortality rate was observed among patients with SARS (86.6%), followed by COVID-19 (76.5%) and MERS (68.5%) (13). However, these findings must be considered in light of various demerits of this study i.e., low quality, published as a research letter, and a small number of primary studies related to SARS and MERS. Another rapid review reported the mortality rate ranging from 10% to 15% in SARS, up to 35% in MERS, and 13% in COVID-19 (56). The low mortality rate among COVID-19 patients as compared to SARS and MERS has also been reported by other studies (30) and might be attributed to the improvement in medical services and facilities through the years, particularly during this pandemic. However, more studies comparing the coronavirus infections are of significant importance during this pandemic.

Only two SRs estimated the pooled incidence of AKI severity stages (4, 46). The variation in the prevalence of AKI stages is attributed to the small number of studies in the synthesis of results. The limited number of primary studies on AKI stages warrants an urgent need for more investigations so severity-based prognosis could be ascertained.

Only 5 studies reported the risk factors for the development of CAKI. Diabetes mellitus and hypertension were most commonly reported risk factors of CAKI in the literature (2, 17, 18, 31), followed by chronic kidney disease (2, 17, 31), cardiovascular disease (2, 17, 31), age (2, 17, 18), and male gender (2, 17, 31). Previous studies (2, 57) have reported that elderly patients with COVID-19 have a more severe or critical illness, comorbid conditions, immunodeficiencies, and secondary infections, rendering them more susceptible to renal injury as compared to younger adults. Moreover, a decrease in the proliferative ability of stem cells in this age group also increases the odds of renal injury (58). Another review also found that patients with age > 60 years had higher odds of developing CAKI as compared to those with age < 60 years (1). Some patients with comorbid conditions such as hypertension and diabetes mellitus are treated with ACE inhibitors (ACEIs) and angiotensin II receptor blockers (ARBs). These drugs upregulate ACE2, thereby increasing the risks of severe or critical COVID-19 infection and the disease severity is well known to be associated with the development of AKI. Elderly patients frequently use these drugs which might be another factor leading to a high incidence of CAKI among them. Lee et al. in their systematic review also reported the RAAS blockade as an independent risk factor of CAKI among COVID-19 patients (38). Available evidence also indicates a great disparity in gender and ethnic distribution of CAKI among patients. Four reviews have identified the male gender as a predictor of CAKI. Recent investigations suggested the role of hormones in the gender-wise pathogenesis of CAKI. The high levels of androgen upregulate the expression of ACE2 and TMPRSSs in renal tissues, as well as modulate the immune response and reduce the antibody response resulting in severe COVID-19 infection. These mechanisms explain the greater risk of CAKI in males (59). Moreover, the expression of ACE2 and TMPRSSs is also higher among elderly people and smokers, making this population more vulnerable to kidney damage (59). The estrogen and progesterone play a protective role by inhibiting SARS-CoV-2 from entering host cells through the downregulation of ACE2 and TMPRSSs, providing a theoretical basis for the lower incidence of CAKI in females (60). These findings indicate that gender differences must be considered while investigating or managing COVID-19 patients. However, the confounding effect of various factors must be adjusted before performing any predictive analysis, i.e., the use of drugs is associated with comorbidities that may be further linked to old age.

This overview found that COVID-19 patients with AKI portend adverse clinical outcomes as compared to those without AKI. The substantial need for RRT has been reported in 16 reviews. These findings indicate that COVID-19 patients with or without AKI require RRT during their management. However, the need for RRT is more among patients with CAKI (47), severe disease (39), admitted to ICU (17), and those with respiratory distress syndrome (ARDS) (28). Furthermore, the patients who died of COVID-19 displayed a higher application rate of RRT than the survival cases (42). It has been reported that critical COVID-19 patients needed RRT within 4 days of their ICU admission. The RRT modalities used were continuous RRT, intermittent hemodialysis and peritoneal dialysis (61). It is important to note that RRT is not only needed to compensate for the renal damage but also required to remove inflammatory factors, thus blocking cytokine storm syndrome among COVID-19 patients and ultimately reducing the damage inflicted on multiple organs (62). However, the indication of RRT, choice of modality, timing of initiation, and duration of use are still some unanswered questions that warrant a dire need for further investigations.

The data pertaining to the mortality due to CAKI were reported in 14 reviews. The AKI-associated mortality in COVID-19 occurred in approximately half of the overall patients and 3/4 of the severe cases and those receiving RRT. It has been noted that CAKI is associated with high mortality even after initiating RRT (11). Cheruiyot et al. reported mortality up to 1/4th of the COVID-19 patients, where severe cases had fourteen times higher chances of death than non-severe cases (14). Despite a wide disparity in fatality rate attributed to CAKI, the results of these reviews explicitly indicate that patients with CAKI had higher odds of mortality than those without kidney injury. It might be due to initiating and perpetuating the cardiac and lung damage due to AKI, underlining the potential multifaceted impact of lung-kidney crosstalk. In this context, the monitoring of kidney functions should be emphasized even among patients with mild illness and particular attention should be paid to those with the compromised renal profile.

## Knowledge gaps and study limitations

Despite a growing body of evidence on CAKI from systematic reviews, the existing literature does not answer some research questions. There is a dearth of investigations presenting the data on AKI staging and its relationship with prognosis, involvement of medications in the pathogenesis of CAKI, and risk factors of CAKI-associated mortality. Moreover, the use of variable definitions of AKI and COVID-19 severity, and the unavailability of baseline serum creatinine may also under- or over- estimate the prevalence of CAKI. In addition, the post-AKI recovery pattern is not assessed in these studies. The data on the management of CAKI and its additional burden on the healthcare budget is not revealed in included SRs. These reviews also did not provide detailed data on CAKI in special populations, i.e., pregnant, cancer, immunodeficient, and pediatric patients.

Since this is a large study intended to summarize and critically appraise the SRs on CAKI, the findings must be interpreted in light of a few shortcomings. This study was not subjected to overlap analysis and some included reviews share various primary studies. However, it is admissible if the purpose of the overview is to summarize the evidence on a specific topic (10). The systematic reviews in this overview may have had some issues as primary studies of these SRs were not checked for the accuracy of data. Some reviews were published as short reports or rapid reviews, thereby providing limited information on search methodology, which may impact the quality assessment of SRs in this overview. However, it is not necessary that the low quality of these SRs is due to poor methodology but might be associated with the lack of reporting. Various SRs in this overview included case series and case reports, thereby impacting the quality of evidence. Furthermore, some SRs also included non-peer-reviewed research studies which were available as preprints at the time of the review process that encourages the critical evaluation of their findings while interpreting the results of this overview. In addition, the results of this overview are combined from moderate to high-quality SRs, thereby indicating the high validity and implications of findings in the clinical practice. However, the use of other quality appraisal tools for systematic reviews may result in variable findings due to the inclusion of studies that we had excluded from this overview. It is important to note that some of the reviews included primary studies classifying AKI with various definitions. The use of different criteria for AKI stratification may result in over- or under-estimation of AKI, thereby, it is suggested to firmly consider the use of the KDIGO criterion to define AKI in future studies. Last but not least, this overview may not cover some studies published after the search date. Nevertheless, this overview carries particular importance and provides an insight into CAKI in a diverse population to policymakers, healthcare professionals, and researchers. This study also revealed some evidence gaps which could help future researchers.

## Conclusion

This overview underscored that AKI is a commonly occurring intricacy among COVID-19 patients. CAKI is associated with higher mortality, severe or critical illness, need for RRT, and poor prognosis. Male gender, old age, certain comorbid conditions (diabetes mellitus, hypertension), smoking, obesity, mechanical ventilation, preexisting renal anomalies, and nephrotoxic drugs are independent risk factors for the development of CAKI. However, this evidence is generated from moderate quality reviews of studies with considerable methodological differences. Despite a large number of SRs, the data on long-term outcomes of CAKI and its association with the severity of renal injury are still scarce. Further investigations should consider these research questions along with the limitations of available evidence so that more firm conclusions can be made. Early detection, timely management including blood purification therapies, adequate hemodynamic support, nephrology consultation, risk assessment through predictive models and avoidance of nephrotoxic drugs may help to improve the vital prognosis of COVID-19 patients with AKI. Since COVID-19 will continue to evolve, it is an impetus to design and implement renal care guidelines for COVID-19 patients.

## Data availability statement

The original contributions presented in this study are included in the article/Supplementary material, further inquiries can be directed to the corresponding author/s.

## Author contributions

TM, YK, AIA, and NA: conceptualization. TM, FK, ASA, MB, and ADA: methodology. YK, ASA, MB, MS, SA, and ZA: software. TM, AIA, NA, and ManA: validation. TM, YK, FK, MB, ADA, MS, and MajA: writing—original draft preparation. TM, YK, AIA, NA, ASA, ZA, and ManA: writing—review and editing. MB: visualization. TM and YK: supervision. TM: project administration. All authors have read and agreed to the published version of the manuscript.
