# Supplementary material for: Incidence, risk factors and outcomes of acute kidney injury among COVID-19 patients: A systematic review of systematic reviews
Source: Front Med (Lausanne). 2022 Nov 4;9:973030. doi: 10.3389/fmed.2022.973030 (PMC9672072; doi:10.3389/fmed.2022.973030)
Supplement: Supplementary file 1 [file Data_Sheet_1.docx]

**Supplementary table 1; PRISMA checklist**

| **Section and Topic** | **Item #** | **Checklist item** | **Location where item is reported** |
| --- | --- | --- | --- |
| **TITLE** | | |  |
| Title | 1 | Identify the report as a systematic review. | 1 |
| **ABSTRACT** | | |  |
| Abstract | 2 | See the PRISMA 2020 for Abstracts checklist. | Supplementary file, page 3 |
| **INTRODUCTION** | | |  |
| Rationale | 3 | Describe the rationale for the review in the context of existing knowledge. | 2 |
| Objectives | 4 | Provide an explicit statement of the objective(s) or question(s) the review addresses. | 2 |
| **METHODS** | | |  |
| Eligibility criteria | 5 | Specify the inclusion and exclusion criteria for the review and how studies were grouped for the syntheses. | 3 |
| Information sources | 6 | Specify all databases, registers, websites, organisations, reference lists and other sources searched or consulted to identify studies. Specify the date when each source was last searched or consulted. | 3 |
| Search strategy | 7 | Present the full search strategies for all databases, registers and websites, including any filters and limits used. | 3 |
| Selection process | 8 | Specify the methods used to decide whether a study met the inclusion criteria of the review, including how many reviewers screened each record and each report retrieved, whether they worked independently, and if applicable, details of automation tools used in the process. | 3 |
| Data collection process | 9 | Specify the methods used to collect data from reports, including how many reviewers collected data from each report, whether they worked independently, any processes for obtaining or confirming data from study investigators, and if applicable, details of automation tools used in the process. | 4 |
| Data items | 10a | List and define all outcomes for which data were sought. Specify whether all results that were compatible with each outcome domain in each study were sought (e.g. for all measures, time points, analyses), and if not, the methods used to decide which results to collect. | 3 |
|  | 10b | List and define all other variables for which data were sought (e.g. participant and intervention characteristics, funding sources). Describe any assumptions made about any missing or unclear information. | 4 |
| Study risk of bias assessment | 11 | Specify the methods used to assess risk of bias in the included studies, including details of the tool(s) used, how many reviewers assessed each study and whether they worked independently, and if applicable, details of automation tools used in the process. | 4 |
| Effect measures | 12 | Specify for each outcome the effect measure(s) (e.g. risk ratio, mean difference) used in the synthesis or presentation of results. | 3 |
| Synthesis methods | 13a | Describe the processes used to decide which studies were eligible for each synthesis (e.g. tabulating the study intervention characteristics and comparing against the planned groups for each synthesis (item #5)). | 7 |
|  | 13b | Describe any methods required to prepare the data for presentation or synthesis, such as handling of missing summary statistics, or data conversions. | 4 |
|  | 13c | Describe any methods used to tabulate or visually display results of individual studies and syntheses. | 4 |
|  | 13d | Describe any methods used to synthesize results and provide a rationale for the choice(s). If meta-analysis was performed, describe the model(s), method(s) to identify the presence and extent of statistical heterogeneity, and software package(s) used. |  |
|  | 13e | Describe any methods used to explore possible causes of heterogeneity among study results (e.g. subgroup analysis, meta-regression). |  |
|  | 13f | Describe any sensitivity analyses conducted to assess robustness of the synthesized results. |  |
| Reporting bias assessment | 14 | Describe any methods used to assess risk of bias due to missing results in a synthesis (arising from reporting biases). |  |
| Certainty assessment | 15 | Describe any methods used to assess certainty (or confidence) in the body of evidence for an outcome. |  |
| **RESULTS** | | |  |
| Study selection | 16a | Describe the results of the search and selection process, from the number of records identified in the search to the number of studies included in the review, ideally using a flow diagram. | 5-26 |
|  | 16b | Cite studies that might appear to meet the inclusion criteria, but which were excluded, and explain why they were excluded. | 5 |
| Study characteristics | 17 | Cite each included study and present its characteristics. | 31-34 |
| Risk of bias in studies | 18 | Present assessments of risk of bias for each included study. |  |
| Results of individual studies | 19 | For all outcomes, present, for each study: (a) summary statistics for each group (where appropriate) and (b) an effect estimate and its precision (e.g. confidence/credible interval), ideally using structured tables or plots. | 19-26 |
| Results of syntheses | 20a | For each synthesis, briefly summarise the characteristics and risk of bias among contributing studies. | 19-26 |
|  | 20b | Present results of all statistical syntheses conducted. If meta-analysis was done, present for each the summary estimate and its precision (e.g. confidence/credible interval) and measures of statistical heterogeneity. If comparing groups, describe the direction of the effect. |  |
|  | 20c | Present results of all investigations of possible causes of heterogeneity among study results. | 19-26 |
|  | 20d | Present results of all sensitivity analyses conducted to assess the robustness of the synthesized results. |  |
| Reporting biases | 21 | Present assessments of risk of bias due to missing results (arising from reporting biases) for each synthesis assessed. |  |
| Certainty of evidence | 22 | Present assessments of certainty (or confidence) in the body of evidence for each outcome assessed. |  |
| **DISCUSSION** | | |  |
| Discussion | 23a | Provide a general interpretation of the results in the context of other evidence. | 27-29 |
|  | 23b | Discuss any limitations of the evidence included in the review. | 29-30 |
|  | 23c | Discuss any limitations of the review processes used. | 29-30 |
|  | 23d | Discuss implications of the results for practice, policy, and future research. | 30 |
| **OTHER INFORMATION** | | |  |
| Registration and protocol | 24a | Provide registration information for the review, including register name and registration number, or state that the review was not registered. | 2 |
|  | 24b | Indicate where the review protocol can be accessed, or state that a protocol was not prepared. | 2 |
|  | 24c | Describe and explain any amendments to information provided at registration or in the protocol. |  |
| Support | 25 | Describe sources of financial or non-financial support for the review, and the role of the funders or sponsors in the review. | 30 |
| Competing interests | 26 | Declare any competing interests of review authors. | 30 |
| Availability of data, code and other materials | 27 | Report which of the following are publicly available and where they can be found: template data collection forms; data extracted from included studies; data used for all analyses; analytic code; any other materials used in the review. | 30 |

| **Section and Topic** | **Item #** | **Checklist item** | **Reported (Yes/No)** |
| --- | --- | --- | --- |
| **TITLE** | | |  |
| Title | 1 | Identify the report as a systematic review. | Yes |
| **BACKGROUND** | | |  |
| Objectives | 2 | Provide an explicit statement of the main objective(s) or question(s) the review addresses. | Yes |
| **METHODS** | | |  |
| Eligibility criteria | 3 | Specify the inclusion and exclusion criteria for the review. | No |
| Information sources | 4 | Specify the information sources (e.g. databases, registers) used to identify studies and the date when each was last searched. | Yes |
| Risk of bias | 5 | Specify the methods used to assess risk of bias in the included studies. | No |
| Synthesis of results | 6 | Specify the methods used to present and synthesise results. | Yes |
| **RESULTS** | | |  |
| Included studies | 7 | Give the total number of included studies and participants and summarise relevant characteristics of studies. | Yes |
| Synthesis of results | 8 | Present results for main outcomes, preferably indicating the number of included studies and participants for each. If meta-analysis was done, report the summary estimate and confidence/credible interval. If comparing groups, indicate the direction of the effect (i.e. which group is favoured). | Yes |
| **DISCUSSION** | | |  |
| Limitations of evidence | 9 | Provide a brief summary of the limitations of the evidence included in the review (e.g. study risk of bias, inconsistency and imprecision). | No |
| Interpretation | 10 | Provide a general interpretation of the results and important implications. | Yes |
| **OTHER** | | |  |
| Funding | 11 | Specify the primary source of funding for the review. | No |
| Registration | 12 | Provide the register name and registration number. | Yes |

**Supplementary Table 2;** Findings extracted from the included Reviews having Low and Criticaly low quality.

| **Authors Years** | **Pooled prevalence /Incidence of CAKI** | **Risk Factors of CAKI**  **(OR / MD / Q / WMD / SMD)** | **Need for RRT** | **CAKI associated mortality or severe disease or other adverse outcomes** | **Comments** |
| --- | --- | --- | --- | --- | --- |
| Chen et al., 2020a [12] | Overall CAKI: 8.9% (20 studies) | NR | NR | NR | The incidence of CAKI is close as in patients with community acquired pneumonia |
| Chen et al., 2020b [13] | NR | NR | NR | Incidence of mortality among CAKI patients: 76.5% (16 studies) | AKI is found as a poor prognosis factor in coronavirus infections, whereby AKI mortality in COVID-19 is higher than MERS but lower than SARS infections |
| Fabrizi et al., 2020 [15] | Summary estimate for overall patients: 0.154  Summary estimate for severe COVID-19 patients: 0.53  Odds ratio of AKI in deceased patients: 15.4 | Meta-regression:  Age (p=0.007)  Arterial hypertension (p=0.001) | Need for RRT:  Overall pooled estimate for the frequency of COVID-19 patients who had AKI and underwent RRT: 0.043 | NR | High heterogeneity and publication biased were observed across studies according to Q, I^2^ and Egger`s regression estimates |
| Lin et al., 2020 [1] | Overall CAKI: 10.6%  CAKI in Non-severe cases: 5.4%  CAKI in Severe: 22.1%  CAKI in Deceased: 22.1% | Age ≥ 60 years (OR): 3.53 (4 studies)  Severe infection (OR): 6.07 (5 studies) | Need for CRRT:  Overall COVID-19: 2.5%  Asia: 1.4%  Europe: 5.4%  North America: 4.0%  Odds for CRRT need in severe COVID-19 (OR): 6.60 (9 studies) | Odds of mortality due to CAKI (OR): 11.05 (5 studies) | The prevalence of CAKI was higher in North America (22.6%) than Europe (11.6%) and Asia (4.5%). The CAKI associated mortality was more in Asia (33.3%) than in Europe (29.4%) and North America (7.4%%). |
| Nogueira et al., 2020 [22] | Incidence of AKI in severe cases: 29% (14 studies)  Incidence of AKI in non-severe cases: 1.2% | NR | NR | NR | CAKI was associated with the high mortality and was more common in severe cases. |
| Vakili et al., 2020 [25] | Overall CAKI: 8.40% (23 studies) | NR | NR | NR | Acute Cardiac Injury and AKI are most frequent non-respiratory complications |
| Yang et al., 2020 [26] | Overall CAKI: 4.5% (17 studies)  Mild or moderation infection: 1.3% (7 studies)  Severe infection: 2.8% (5 studies)  Critical infection: 36.4% (7 studies)  Non-survivors: 52.9%  Survivors: 0.7% | NR | Need of CRRT:  Severe patients: 5.6% (3 studies)  Non-severe patients: 0.1% (3 studies) | CAKI associated mortality: 2.9% (3 studies) | The renal involvement during COVID-19 varied from abnormal urine analysis, elevated SCr or BUN levels, to AKI and renal failure for which CRRT is required |
| Zhang et al., 2020 [27] | NR | Male (OR): 3.43 (4 studies)  DM (OR): 2.63 (4 studies)  COPD (OR): 2.98 (3 studies)  CVD (OR): 2.26 (3 studies)  CKD (OR): 3.26 (3 studies)  Cerebrovascular disease: 2.95 (3 studies) | NR | Odds of mortality due to CAKI (OR): 8.78 (3 studies) | All the studies were from China, selection bias |
| Brienza et al., 2021 [29] | Overall CAKI: 18.3% (10 studies)  Severe COVID-19 patients: 20% (5 studies) | NR | Need of CRRT in COVID-19: 2.4%  Need of CRRT in severe COVID-19: 7.5% (5 studies) | Mortality rate among patients with CAKI: 52% (5 studies)  Odds of mortality due to CAKI (OR): 31.03 (5 studies) | CAKI is associated with increased need for CRRT and mortality. |
| Cau et al., 2021 [31] | Critically ill patients group C: 51% (71 studies)  Critically ill patients group A: 52% (21 studies)  Critically ill patients in group non-A: 63% (3 studies)  Critically ill patients group C: 52% with KDIGO criteria (61 studies)  Group C: critically ill COVID-19 patients  Group A: patients infected with ACE2-associated viruses  Group non-A: non-ACE2 associated viruses | NR | RRT frequency  Critically ill patients group C: 20%  Critically ill patients group A: 18%  Critically ill patients in group non-A: 49%  Critically ill patients group C: 22% with KDIGO criteria (61 studies) | Mortality  Both critical and non-critical COVID-19 patients: 42%  Shock  Critically ill patients group C: 24%  Critically ill patients group A: 25%  Critically ill patients in group non-A: 78%  Vasopressor use  Critically ill patients group C: 26%  Critically ill patients group A: 44%  Critically ill patients in group non-A: 78% | AKI frequency did not significantly differ between three virus groups (C, A, non-A)  RRT frequency did not significantly differ between C and A groups  RRT frequency of C and A group significantly differ from non-A group  Shock and vasopressor use was significantly associated with AKI (OR: 1.31) and RRT use (OR: 1.36)  Mortality associated with COVID-19 AKI is lower than that associated with MERS AKI (81%) and SARS AKI (90%) |
| Chan et al., 2021b [33] | Overall CAKI: 7.58 (12 studies) | NR | NR | Mortality rate in CAKI: 93.27% (3 studices) | Most of the studies were from China (n=22) |
| Daniella, 2021 [36] | Overall CAKI: 36.44% (4 studies) | Qualitative synthesis of risk factors  African American race (2 studies)  Age (2 studies)  CKD (1 study)  DM (1 study)  CVD [CAD, HF, PVD] (1 study)  HTN (1 study)  HPL (1 study)  PCT elevated (1 study)  eGFR < 60 ml/min (1 study)  Higher sofa score at admission (1 study)  MV (1 study)  Vasoactive medication use (1 study) | NR | NR | Prothrombotic state of COVID-19 is also associated with the development of CAKI. |
| Menon et al., 2021 [41] | Overall CAKI:11% (20 studies) | Severe COVID-19 infection (OR): 8.45 (12 studies) | NR | Odds of mortality among patients with CAKI (OR): 13.52 (8 studies) | High heterogeneity among studies from which AKI incidence was estimated, while heterogeneity was negligible in Meta regression |
| Oliveira, 2021 [42] | Overall CAKI: 12.3% (21 studies)  Incidence of CAKI, after removal of 6 outlier studies 11% (15 studies)  Prevalence of AKI in critically ill patients  77.3% (21 studies) and 80.6% (20 studies, after removal of 1 outlier study) | Older age: only in univariate meta-regression analysis (p=0.005) – Odds ratio is not reported  All multivariate meta-regression analyses did not reveal association of CAKI with any variable | Need of RRT CAKI: 23.4% (21 studies) and 26.4% (20 studies, after removal of 1 outlier study) | Mortality rate among CAKI cases: 67% (21 studies) and 75.7% (20 studies, after removal of 1 outlier study)  Odds of mortality due to CAKI (OR): 13.3 (8 studies) | Heterogeneity among samples were high therefore all analyses |
| Passoni et al., 2021 [44] | Overall CAKI: 9.2% (28 studies)  In ICU: 32.6% (15 studies)  In USA: 22.9% (4 studies)  In elderly patients: 22.9% (2 studies)  Early hospitalization: 11.9% (20 studies)  In ARDS: 4.3% (21 studies)  In ACI: 9.3% (2 studies)  In secondary infection: 31.6% (studies)  Sepsis/shock: 4.7% (15 studies) | NR | Need for RRT: 3.2% (17 studies) | Mortality rate among patients with CAKI: 50.4% (9 studies) | 2 out 30 studies reported no AKI in COVID-19 patients |
| Raina et al., 2021a [45] | Overall CAKI: 19.45% (53 studies) | Chines studies: 2.34  Prospective/retrospective study design: 1.80  Studies with mean/median age ≥60 years (OR): 3.87 | Need of KRT in overall COVID-19 patients: 8.83% (33 studies)  Need of KRT in CAKI: 39.04% (4 studies) | Prevalence of Morality in CAKI: 54.25% (13 studies)  Prevalence of Morality in COVID-19: 19.35% (25 studies)  Odds of mortality in CAKI (OR): 18.63 (5 studies) | CAKI was observed as negative prognostic factor among COVID-19 patients. |
| Yang et al., 2021 [49] | Overall CAKI: 12.3% (51 studies)  Transplant patients: 38.9% (8 studies)  Deceased cases: 42% (12 studies)  Asia: 6.9 (32 studies)  North America: 34.6 (8 studies)  Europe: 22.9 (10 studies)  China: 6.5 (29 studies)  ICU: 39% (13)  KDIGO staging (7 studies)  S1: 36.3%  S2: 20.7%  S3: 43% | R | Need of RRT  Overall COVID-19: 5.4% (39 studies)  Transplant patents: 15.6% (5 Studies)  ICU patients: 16.3% (12 studies) | NR | Most of the studies were from China, thereby yielding potential selection bias |
| *Abbreviations*: ACE: angiotensin converting enzyme, ACEIs: angiotensin converting enzyme inhibitors, ACI: acute cardiac injury, AKI: acute kidney injury, ARBs: angiotensin receptor blockers, ARDS: acute respiratory distress syndrome, BUN: blood Urea Nitrogen, CAD: Coronary artery disease, CAKI: COVID-19 associated AKI, CKD: chronic kidney disease, COPD: chronic obstructive pulmonary disease, COVID-19: Corona Virus Disease 19, CRRT: continuous renal replacement therapy, CVD: cardiovascular disease, DM: diabetes Mellitus, eGFR: estimated glomerular filtration rate, HF: Heart Failure, HPL: Hyperlipidemia, HTN: Hypertension, ICU: intensive care unit, ICU: intensive Care Unit, KRT: kidney replacement therapy, KTRs: kidney Transplant recipients, MERS: Middle East Respiratory Syndrome, MV: mechanical ventilation, NOS: Newcastle-Ottawa Scale, NR: not reported, PCT: procalcitonin, PVD: peripheral vascular disease, RAAS: Renin angiotensin aldosterone system, RR: risk ratio, RRT: renal replacement therapy, RRT: renal replacement therapy, SARS: Severe acute respiratory syndrome, SCr: serum creatinine, SMD: standardized mean difference, WMD: weighted mean difference | | | | | |

**Search strategy**

***ProQuest (n=29)***

ti(Acute Kidney Injury) OR ti(acute failure renal) OR ti(Renal Failure) OR ti(renal impairment) OR ti(kidney impairment) OR ti(cause of kidney failure) AND ti(SARS-CoV-2) AND ti(COVID-19) AND ti(Coronavirus Disease) AND ti(Novel Coronavirus) [Filters: Scholarly Journals, Last 5 Years, Correspondence OR Commentary OR Letter To The Editor OR Literature Review, English]

***SCOPUS (n=1857)***

TITLE-ABS-KEY ( "COVID-19" OR "2019-nCoV" OR "SARS-CoV-2" OR "COVID-19" OR "Coronavirus Disease" OR " Novel Coronavirus" ) AND ( "Acute Kidney Injury" OR "Acute kidney injury" OR "renal failure" OR "acute renal injury" OR "acute kidney failure" OR "Kidney injury" OR "renal impairment" OR "kidney impairment" ) AND ( "Systematic Review" OR "review" OR "systematic review" OR "meta-analysis" OR "meta-regression" ) AND ( LIMIT-TO ( PUBYEAR , 2022 ) OR LIMIT-TO ( PUBYEAR , 2021 ) OR LIMIT-TO ( PUBYEAR , 2020 ) OR LIMIT-TO ( PUBYEAR , 2019 ) ) AND ( LIMIT-TO ( DOCTYPE , "re" ) ) AND ( LIMIT-TO ( LANGUAGE , "English" ) ) AND ( LIMIT-TO ( SRCTYPE , "j" ) )

***Prospero Register (n=1243)***

1243 records found for * AND (Review_Completed_not_published OR Review_Completed_published):RS AND (covid-19):HA

***PubMed (n=479)***

***Concept 1: COVID-19***

**Keywords**: "COVID-19"[Mesh] OR “2019-nCoV”[tw] OR “SARS-CoV-2”[tw] OR “COVID-19”[tw] OR “Coronavirus Disease”[tw] OR “ Novel Coronavirus”[tw]

**MeSH**: "COVID-19"[Mesh]

***Concept 2: Acute Kidney Injury***

**Keywords**: “Acute Kidney Injury"[Mesh] OR “Acute kidney injury”[tw] OR “acute renal failure”[tw] OR “acute renal injury”[tw] OR “acute kidney failure”[tw] OR “Kidney injury”[tw] OR “renal impairment”[tw] OR “kidney impairment”[tw]

**MeSH**: "Acute Kidney Injury"[Mesh]

***Concept 3: Systematic Review***

**Keywords**: "Systematic Review"[Publication Type] OR “review”[tw] OR “systematic review”[tw] OR “meta-analysis”[tw] OR “meta-regression”[tw]

**MeSH**: "Systematic Review" [Publication Type]

| **#**1 | "COVID-19"[Mesh] OR “2019-nCoV”[tw] OR “SARS-CoV-2”[tw] OR “COVID-19”[tw] OR “Coronavirus Disease”[tw] OR “ Novel Coronavirus”[tw] |
| --- | --- |
| **#**2 | “Acute Kidney Injury"[Mesh] OR “Acute kidney injury”[tw] OR “acute renal failure”[tw] OR “acute renal injury”[tw] OR “acute kidney failure”[tw] OR “Kidney injury”[tw] OR “renal impairment”[tw] OR “kidney impairment”[tw] |
| **#**3 | "Systematic Review"[Publication Type] OR “review”[tw] OR “systematic review”[tw] OR “meta-analysis”[tw] OR “meta-regression”[tw] |
| **#**4 | #1 AND #2 AND #3 |

**Combined Concepts by Text Word [tw]**

("COVID-19"[MeSH Terms] OR "2019-nCoV"[Text Word] OR "SARS-CoV-2"[Text Word] OR "COVID-19"[Text Word] OR "Coronavirus Disease"[Text Word] OR "Novel Coronavirus"[Text Word]) AND ("acute kidney injury"[MeSH Terms] OR "acute kidney injury"[Text Word] OR "acute renal failure"[Text Word] OR "acute renal injury"[Text Word] OR "acute kidney failure"[Text Word] OR "Kidney injury"[Text Word] OR "renal impairment"[Text Word] OR "kidney impairment"[Text Word]) AND ("Systematic Review"[Publication Type] OR "review"[Text Word] OR "Systematic Review"[Text Word] OR "meta-analysis"[Text Word] OR "meta-regression"[Text Word])

***PMC (n=271)***

| **#**1 | (((COVID-19[Title] OR (Novel[Title] AND Coronavirus[Title])) OR 2019-nCoV[Title]) OR SARS-CoV-2[Title]) OR (Coronavirus[Title] AND Disease[Title]) |
| --- | --- |
| **#**2 | ((((((Acute[Title] AND Kidney[Title] AND Injury[Title]) OR (Kidney[Title] AND injury[Title])) OR (acute[Title] AND renal[Title] AND failure[Title])) OR (acute[Title] AND renal[Title] AND injury[Title])) OR (acute[Title] AND kidney[Title] AND failure[Title])) OR (renal[Title] AND impairment[Title])) OR (kidney[Title] AND impairment[Title]) |
| **#**3 | (((Systematic[Title] AND Review[Title]) OR review[Title]) OR meta-analysis[Title]) OR meta-regression[Title] |
| **#**1 + **#**2 + **#**3 | ((#1) AND #2) AND #3 |

Combined Concept by TITLE (n=46)

(((((((COVID-19) OR Novel Coronavirus) OR 2019-nCoV) OR SARS-CoV-2) OR Coronavirus Disease)) AND (((((((Acute Kidney Injury) OR Kidney injury) OR acute renal failure) OR acute renal injury) OR acute kidney failure) OR renal impairment) OR kidney impairment)) AND ((((Systematic Review) OR review) OR meta-analysis) OR meta-regression)

Combined Concept by Abstract (n=225)

(((((COVID-19[Abstract] OR Novel Coronavirus[Abstract]) OR 2019-nCoV[Abstract]) OR SARS-CoV-2[Abstract]) OR Coronavirus Disease[Abstract]) AND ((((((Acute Kidney Injury[Abstract] OR Kidney injury[Abstract]) OR acute renal failure[Abstract]) OR acute renal injury[Abstract]) OR acute kidney failure[Abstract]) OR renal impairment[Abstract]) OR kidney impairment[Abstract])) AND (((Systematic Review[Abstract] OR review[Abstract]) OR meta-analysis[Abstract]) OR meta-regression[Abstract])

***CENTRAL (n=0)***

ID Search Hits

#1 MeSH descriptor: [COVID-19] explode all trees 1722

#2 MeSH descriptor: [Acute Kidney Injury] explode all trees 1544

#3 MeSH descriptor: [Review] explode all trees 2

#4 (COVID-19 NEXT (disease* or infection*)):ti,ab,kw OR (SARS-CoV-2):ti,ab,kw OR (Coronavirus NEXT (disease* or infection*)):ti,ab,kw OR (Novel NEXT (Coronavirus*)):ti,ab,kw OR ("SARS-CoV"):ti,ab,kw (Word variations have been searched) 7728

#5 ("acute renal failure"):ti,ab,kw OR (acute NEXT (injury* or failure*)):ti,ab,kw OR ("renal failure"):ti,ab,kw OR ("kidney failure"):ti,ab,kw AND ("renal impairment"):ti,ab,kw (Word variations have been searched) 7675

#6 ("systematic review"):ti,ab,kw OR ("meta analysis"):ti,ab,kw OR ("meta-regression"):ti,ab,kw AND ("meta analyses"):ti,ab,kw AND ("systematic"):ti,ab,kw (Word variations have been searched) 26618

#7 #1 AND #2 AND #3 0

#8 #4 AND #5 AND #6 0

| Cochrane Reviews | Cochrane Protocols | Trials | Editorials | Special collections | Clinical Answers | Other evidence |
| --- | --- | --- | --- | --- | --- | --- |
| 0 | 0 | 0 | 0 | 0 | 0 | 0 |
